# Supplementary material for: Genomic diversity and global distribution of four new prasinoviruses from the tropical north Pacific
Source: Microbiol Spectr. 2025 Oct 8;13(11):e02583-24. doi: 10.1128/spectrum.02583-24 (PMC12584671; doi:10.1128/spectrum.02583-24)
Supplement: Supplemental figures and tables — Tables S1 to S3 and Fig. S1 to S6. [file spectrum.02583-24-s0001.docx]

Supplementary Materials

**Supplementary Table S1.** Antibiotic recipe used to clean *Micromonas* culture of bacteria and associated phage. This recipe was developed by colleagues at Observatoire océanologique de Banyuls-sur-Mer.

| **Antibiotic name** | **Final concentration (µg/mL)** | **Mass (g) in 1000X stock (10mL)** |
| --- | --- | --- |
| Ampicillin | 50 | 0.5 |
| Gentamicin | 50 | 0.5 |
| Kanamycin | 20 | 0.2 |
| Neomycin | 100 | 1 |

**Supplementary Table S2.** Strain information for prasinovirus and chlorovirus strains used in OrthoFinder and phylogenetic analysis.

| **Accession** | **Strain Name** | **Strain Abbreviation** | **Host Genus** | **Authors** |
| --- | --- | --- | --- | --- |
| HM004430 | Bathycoccus sp. RCC1105 virus | BpV2 | Bathycoccus | Moreau et al. (2010) |
| HM004432 | Bathycoccus sp. RCC1105 virus | BpV1 | Bathycoccus | Moreau et al. (2010) |
| MK522034 | Bathycoccus sp. RCC716 virus 1 | BII-V1 | Bathycoccus | Bachy et al. (2019) |
| MK522038 | Bathycoccus sp. RCC716 virus 2 | BII-V2 | Bathycoccus | Bachy et al. (2019) |
| MK522039 | Bathycoccus sp. RCC716 virus 3 | BII-V3 | Bathycoccus | Bachy et al. (2019) |
| HQ633072 | Micromonas pusilla virus PL1 | MpV-PL1 | Micromonas | Henn et al. (2010) |
| JF974320 | Micromonas pusilla virus SP1 | MpV-SP1 | Micromonas | Henn et al. (2010) |
| NC_014767 | Micromonas sp. RCC1109 virus | MpV1 | Micromonas | Moreau et al. (2010) |
| NC_020864 | Micromonas pusilla virus 12T | MpV-12T | Micromonas | Henn et al. (2010) |
| HQ633059 | Ostreococcus lucimarinus virus 6 | OlV6 | Ostreococcus | Henn et al. (2010) |
| HQ633060 | Ostreococcus lucimarinus virus 3 | OlV3 | Ostreococcus | Henn et al. (2010) |
| JF974316 | Ostreococcus lucimarinus virus 4 | OlV4 | Ostreococcus | Henn et al. (2010) |
| MK514405 | Ostreococcus lucimarinus virus 1 | OlV1 | Ostreococcus | Zimmerman et al. (2019) |
| MK514406 | Ostreococcus lucimarinus virus 7 | OlV7 | Ostreococcus | Zimmerman et al. (2019) |
| NC_020852 | Ostreococcus lucimarinus virus 5 | OlV5 | Ostreococcus | Henn et al. (2010) |
| NC_028091 | Ostreococcus lucimarinus virus 2 | OlV2 | Ostreococcus | Derelle et al. (2015) |
| NC_028092 | Ostreococcus mediterraneus virus 1 | OmV1 | Ostreococcus | Derelle et al. (2015) |
| EU304328 | Ostreococcus tauri virus 5 | OtV5 | Ostreococcus | Derelle et al. (2015) |
| FN386611 | Ostreococcus tauri virus 1 | OtV1 | Ostreococcus | Weynberg et al. (2009) |
| FN600414 | Ostreococcus tauri virus 2 | OtV2 | Ostreococcus | Weynberg et al. (2009) |
| JN225873 | Ostreococcus tauri virus RT-2011 | OtV-RT2011 | Ostreococcus | Thomas et al. (2011) |
| DQ491002 | Paramecium bursaria Chlorella virus NY2A | NY2A | Paramecium bursaria Chlorella | Fitzgerald et al. (2007) |
| DQ491003 | Paramecium bursaria Chlorella virus AR158 | AR158 | Paramecium bursaria Chlorella | Fitzgerald et al. (2007) |
| DQ890022 | Paramecium bursaria Chlorella virus FR483 | FR483 | Paramecium bursaria Chlorella | Fitzgerald et al. (2007) |
| NC_000852 | Paramecium bursaria Chlorella virus 1 | PbCV1 | Paramecium bursaria Chlorella | Yanai-Balser et al. (2010) |
|  |  |  |  |  |

**Supplementary Table S3**. Orthogroups that exhibit highly significant differences between viruses infecting different host genera (p < 0.001), and which possess putative functional annotations. Reported for each orthogroup is the p-value (adjusted for false discovery rate) from a chi-square likelihood ratio test comparing occurrence across host genera, the organism(s) associated with the top refseq_protein database hits, selected annotations from refseq_protein and InterPro, and the proportion of strains infecting a specific host genus that have sequences present in the orthogroup.

| **Orthogroup** | **Adjusted p-value** | **Organisms** | **Selected Annotations** | **Proportion *Bathycoccus-* infecting virus strains** | **Proportion *Micromonas*- infecting virus strains** | | **Proportion *Ostreococcus*- infecting virus strains** |
| --- | --- | --- | --- | --- | --- | --- | --- |
| OG0000005 | 7.12E-06 | Bathycoccus sp. RCC1105 virus BpV1 | Intramolecular chaperone auto-processing domain, Galactose oxidase/kelch, Integrin alpha beta-propellor, FG-GAP repeat | 0.80 | 0.00 | 0.38 | |
| OG0000008 | 0.00047661 | *Micromonas* sp. RCC1109 virus MpV1, *Micromonas* pusilla virus SP1, Ancylomarina sp. DW003 | Serralysin-like metalloprotease, C-terminal / Tumour necrosis factor-like domain superfamily, C1q domain | 0.20 | 1.00 | | 0.88 |
| OG0000016 | 5.26E-06 | *Micromonas* pusilla virus 12T, *Micromonas* sp. RCC1109 virus MpV1 | Coagulation factor 5/8, Concanavalin A-like lectin, Galactose oxidase, Integrin alpha, Serralysin-like metalloprotease, Tumour necrosis factor-like domain superfamily | 0.20 | 0.00 | | 0.88 |
| OG0000135 | 1.13E-06 | *Micromonas* pusilla virus 12T | NFACT, RNA-binding domain | 0.00 | 1.00 | | 0.50 |
| OG0000141 | 1.65E-19 | *Micromonas* sp. RCC1109 virus MpV1,*Micromonas* pusilla virus 12T | Asparagine synthetase | 0.00 | 1.00 | | 0.88 |
| OG0000142 | 52E-05 | *Micromonas* pusilla virus 12T, *Micromonas* sp. RCC1109 virus MpV1, *Micromonas* pusilla virus SP1 | dCMP deaminase | 0.00 | 0.67 | | 1.00 |
| OG0000143 | 4.69E-05 | Pseudodesulfovibrio sp. SB368, *Micromonas* pusilla virus SP1, *Micromonas* pusilla virus 12T, Ostreococcus tauri virus 1, Chromobacterium amazonense | tail fiber protein | 0.00 | 0.25 | | 0.88 |
| OG0000144 | 15E-05 | *Micromonas* sp. RCC1109 virus MpV1, *Micromonas* pusilla virus SP1 | cAMP-dependent Kef-type K+ transporter | 0.60 | 0.17 | | 1.00 |
| OG0000149 | 0.00024067 | *Micromonas* pusilla virus SP1 | methyltransferase | 0.00 | 1.00 | | 0.63 |
| OG0000157 | 0 | Ostreococcus tauri virus 1, Ostreococcus lucimarinus virus 1, Ostreococcus lucimarinus virus 7, *Micromonas* sp. RCC1109 virus MpV1 | NTP pyrophosphohydrolase MazG-related, YvdC | 0.00 | 1.00 | | 1.00 |
| OG0000159 | 1.04E-08 | *Micromonas* sp. RCC1109 virus MpV1 | Rhodanese-like domain superfamily | 0.00 | 1.00 | | 0.63 |
| OG0000163 | 1.65E-19 | Ostreococcus mediterraneus virus 1, *Micromonas* sp. RCC1109 virus MpV1, Ostreococcus lucimarinus virus OlV5 | Nucleotide-diphospho-sugar transferases | 0.00 | 1.00 | | 0.88 |
| OG0000168 | 52E-05 | *Micromonas* pusilla virus 12T, *Micromonas* sp. RCC1109 virus MpV1 | DNA polymerase beta-like, N-terminal domain | 0.00 | 0.67 | | 1.00 |
| OG0000174 | 1.05E-05 | *Micromonas* sp. RCC1109 virus MpV1 | Holliday junction resolvase, A22, Ribonuclease H-like superfamily | 0.00 | 0.92 | | 0.75 |
| OG0000181 | 4.07E-07 | *Micromonas* pusilla virus 12T, *Micromonas* pusilla virus SP1 | endonuclease | 1.00 | 0.25 | | 1.00 |
| OG0000183 | 1.97E-06 | Acinetobacter larvae | lipid A hydroxylase LpxO | 0.20 | 1.00 | | 0.25 |
| OG0000187 | 1.71E-12 | Suillus clintonianus, *Micromonas* pusilla virus 12T | ubiquitin | 0.00 | 0.00 | | 0.88 |
| OG0000213 | 0.00017211 | *Micromonas* pusilla virus SP1 | cytidyltransferase | 0.00 | 0.83 | | 0.25 |
| OG0000227 | 5.32E-05 | *Micromonas* pusilla virus 12T | Zinc finger C2M2-type | 0 | 0.83 | | 0.75 |
| OG0000251 | 49E-09 | *Micromonas* sp. RCC1109 virus MpV1;*Micromonas* pusilla virus SP1 | mannitol dehydrogenase | 0.00 | 0.08 | | 0.88 |
| OG0000300 | 49E-09 | *Micromonas* sp. RCC1109 virus MpV1;*Micromonas* pusilla virus SP1 | Glycerophosphodiester phosphodiesterase domain, PLC-like phosphodiesterase, TIM beta/alpha-barrel domain superfamily | 0.00 | 0.08 | | 0.88 |
| OG0000467 | 3.26E-05 | *Micromonas* pusilla virus 12T | Glucose-6-phosphate dehydrogenase, NAD(P)-binding domain superfamily | 0.00 | 0.00 | | 0.63 |

**Supplementary Table S4.** Full metagenomic dataset searched with CoverM, including Sequence Read Archive (HOT and GEOTRACES) and European Read Archive (Tara Oceans) accession numbers and metadata. The spreadsheet can be found online [here](https://docs.google.com/spreadsheets/d/1kR85LzDAh1uXZi9xIoilpY7GRiLLtOugO6wFpkfjNmk/edit?usp=sharing) and through the attached csv file.

**Supplementary Table S5.** Orthogroups found in all four HiMcVs (i.e., core HiMcV orthogroups). Table includes top hits from refseq_protein BLAST, information from InterPro member databases, and HiMcV putative gene IDs. The spreadsheet can be found [here](https://docs.google.com/spreadsheets/d/1tPO_nPqjQ0H_sf4tIiTADOLBXLn-mzUshCn5cDruZAg/edit#gid=0) and through the attached csv file.

**Supplementary Table S6.** Orthogroups not shared by all four HiMcVs (i.e., non-core HiMcV orthogroups), including those not found in other prasinoviruses. Table includes top hits from refseq_protein BLAST, GOEV, information from InterPro member databases, and HiMcV putative gene IDs. Unique orthogroups are highlighted in yellow. The spreadsheet can be found [here](https://docs.google.com/spreadsheets/d/1pn6YQXgFvQT3EVGtD95_CerW9_MoKgWE51bj5CSzAps/edit?usp=sharing) and through the attached csv file.

**Supplementary Table S7.** Orthogroups shared between HiMcVs and *Micromonas* hosts M1 and M2 Table includes top hits for HiMcVs from refseq_protein BLAST, information from InterPro member databases, HiMcV putative gene IDs, as well as the numbers of host and HiMcV strains with sequences present in each orthogroup. The spreadsheet can be found [here](https://docs.google.com/spreadsheets/d/1kq_7FP1AqZxjLFNAg7cp8nfCbIg8uDlBBItUHzOuDOU/edit?usp=sharing) and through the attached csv file.

**Supplementary Table S8.** Comparison of orthogroup occurrence across viruses infecting different host genera. Columns for each prasinovirus strain contain sequence count data for each orthogroup (i.e., values > 1 indicate multiple paralogs per strain). Raw p-values, p-values adjusted for false discovery rates, and sequence annotation are included. The corresponding spreadsheet can be found [here](https://docs.google.com/spreadsheets/d/1n-t7b3mGLXKZa8vqilt-RS8bHSN4rTjGy51ncbfG0vo/edit?usp=sharing) and through the attached csv file.

**Supplementary Table S9.** Metagenome samples containing reads that mapped successfully to HiMcV assemblies, using the CoverM criteria of 95% nucleotide identity and 20% cover. Table includes strain name of virus, GenBank NCBI SRA or European Read Archive accessions, percent of unmapped reads from each run, relative abundance of reads mapping to HiMcV assembly, and the name of the metagenomic data set. The BIOGEOTRACES (BGT) dataset are from Biller *et al*. (2018),the Hawaii Ocean Timeseries (HOT) data set is from Mende *et al*. (2017), and the Tara data set is from Brum *et al*. (2015).

| **Genome** | **SRA/ENA_Accession** | **unmapped(%)** | **RelativeAbundance(%)** | **DataSet** |
| --- | --- | --- | --- | --- |
| **McVKB2** | SRR5788033 | 99.9834 | 0.00304499 | BGT |
| **McVKB2** | SRR5788075 | 99.98971 | 0.00126892 | BGT |
| **McVKB2** | SRR5788032 | 99.984024 | 0.0026116 | BGT |
| **McVKB2** | SRR5788130 | 99.973625 | 0.0019791 | BGT |
| **McVKB2** | SRR5788131 | 99.98399 | 0.00956761 | BGT |
| **McVKB2** | SRR5788207 | 99.970116 | 0.00395817 | BGT |
| **McVKB2** | SRR5788208 | 99.96208 | 0.00564524 | BGT |
| **McVKB2** | SRR5788209 | 99.96813 | 0.00430875 | BGT |
| **McVKB2** | SRR5788210 | 99.97286 | 0.00374107 | BGT |
| **McVKB2** | SRR5788211 | 99.96458 | 0.00538717 | BGT |
| **McVKB2** | SRR5788212 | 99.97119 | 0.00460325 | BGT |
| **McVKB2** | ERR594376 | 99.99707 | 0.00293018 | Tara |
| **McVKB2** | ERR594392 | 99.965965 | 0.00068961 | Tara |
| **McVKB2** | ERR594414 | 99.921646 | 0.00219858 | Tara |
| **McVKB2** | ERR594382 | 99.92059 | 0.00213045 | Tara |
| **McVKB2** | ERR594361 | 99.9567 | 0.0013392 | Tara |
| **McVKB2** | ERR594359 | 99.95748 | 0.00133211 | Tara |
| **McVKB2** | ERR594389 | 99.98083 | 0.0013314 | Tara |
| **McVKB2** | ERR594362 | 99.98643 | 0.00087922 | Tara |
| **McVKB2** | ERR599350 | 99.99125 | 0.00324052 | Tara |
| **McVKB3** | SRR5788033 | 99.9834 | 0.00474699 | BGT |
| **McVKB3** | SRR5788075 | 99.98971 | 0.00126892 | BGT |
| **McVKB3** | SRR5788026 | 99.98261 | 0.00454977 | BGT |
| **McVKB3** | SRR5788027 | 99.98899 | 0.00335733 | BGT |
| **McVKB3** | SRR5788028 | 99.994484 | 0.00177133 | BGT |
| **McVKB3** | SRR5788030 | 99.99456 | 0.00183901 | BGT |
| **McVKB3** | SRR5788031 | 99.99267 | 0.0025355 | BGT |
| **McVKB3** | SRR5788032 | 99.984024 | 0.00448607 | BGT |
| **McVKB3** | SRR5788130 | 99.973625 | 0.00782666 | BGT |
| **McVKB3** | SRR5788131 | 99.98399 | 0.0028609 | BGT |
| **McVKB3** | SRR5788136 | 99.98759 | 0.00442311 | BGT |
| **McVKB3** | SRR5788137 | 99.98548 | 0.00462654 | BGT |
| **McVKB3** | SRR5788207 | 99.970116 | 0.00713887 | BGT |
| **McVKB3** | SRR5788208 | 99.96208 | 0.00944147 | BGT |
| **McVKB3** | SRR5788209 | 99.96813 | 0.00766576 | BGT |
| **McVKB3** | SRR5788210 | 99.97286 | 0.00671979 | BGT |
| **McVKB3** | SRR5788211 | 99.96458 | 0.00913204 | BGT |
| **McVKB3** | SRR5788212 | 99.97119 | 0.00693099 | BGT |
| **McVKB3** | ERR594385 | 99.995705 | 0.00150004 | Tara |
| **McVKB3** | ERR594392 | 99.965965 | 0.02087303 | Tara |
| **McVKB3** | ERR594414 | 99.921646 | 0.04176986 | Tara |
| **McVKB3** | ERR594382 | 99.92059 | 0.04229004 | Tara |
| **McVKB3** | ERR594361 | 99.9567 | 0.02593655 | Tara |
| **McVKB3** | ERR594359 | 99.95748 | 0.02555807 | Tara |
| **McVKB3** | ERR594389 | 99.98083 | 0.01358112 | Tara |
| **McVKB3** | ERR594362 | 99.98643 | 0.00969066 | Tara |
| **McVKB3** | ERR599374 | 99.99579 | 0.00303531 | Tara |
| **McVKB4** | ERR594414 | 99.921646 | 0.00327259 | Tara |
| **McVKB4** | ERR594382 | 99.92059 | 0.00331005 | Tara |
| **McVKB4** | ERR599350 | 99.99125 | 0.00218787 | Tara |
| **McVSA1** | SRR5720231 | 99.99596 | 0.00404624 | BGT |
| **McVSA1** | SRR5720232 | 99.999 | 0.00100338 | BGT |
| **McVSA1** | SRR5720236 | 99.99674 | 0.00325706 | BGT |
| **McVSA1** | SRR5720237 | 99.99721 | 0.00279628 | BGT |
| **McVSA1** | SRR5720238 | 99.99823 | 0.00177218 | BGT |
| **McVSA1** | SRR5720249 | 99.99895 | 0.00104743 | BGT |
| **McVSA1** | SRR5720250 | 99.99886 | 0.00114006 | BGT |
| **McVSA1** | SRR5720254 | 99.99887 | 0.00113518 | BGT |
| **McVSA1** | SRR5720256 | 99.999146 | 0.00085947 | BGT |
| **McVSA1** | SRR5720259 | 99.99889 | 0.00110498 | BGT |
| **McVSA1** | SRR5788033 | 99.9834 | 0.00880504 | BGT |
| **McVSA1** | SRR5788075 | 99.98971 | 0.00126892 | BGT |
| **McVSA1** | SRR5788089 | 99.99724 | 0.00276746 | BGT |
| **McVSA1** | SRR5788109 | 99.99846 | 0.00154367 | BGT |
| **McVSA1** | SRR5788026 | 99.98261 | 0.0128368 | BGT |
| **McVSA1** | SRR5788027 | 99.98899 | 0.0076518 | BGT |
| **McVSA1** | SRR5788028 | 99.994484 | 0.00374259 | BGT |
| **McVSA1** | SRR5788030 | 99.99456 | 0.00360023 | BGT |
| **McVSA1** | SRR5788031 | 99.99267 | 0.00479299 | BGT |
| **McVSA1** | SRR5788032 | 99.984024 | 0.00887816 | BGT |
| **McVSA1** | SRR5788130 | 99.973625 | 0.01656745 | BGT |
| **McVSA1** | SRR5788131 | 99.98399 | 0.00357325 | BGT |
| **McVSA1** | SRR5788136 | 99.98759 | 0.00798864 | BGT |
| **McVSA1** | SRR5788137 | 99.98548 | 0.00989475 | BGT |
| **McVSA1** | SRR5788207 | 99.970116 | 0.01878805 | BGT |
| **McVSA1** | SRR5788208 | 99.96208 | 0.02283623 | BGT |
| **McVSA1** | SRR5788209 | 99.96813 | 0.01989256 | BGT |
| **McVSA1** | SRR5788210 | 99.97286 | 0.01668005 | BGT |
| **McVSA1** | SRR5788211 | 99.96458 | 0.02090218 | BGT |
| **McVSA1** | SRR5788212 | 99.97119 | 0.01727143 | BGT |
| **McVSA1** | SRR5788229 | 99.99854 | 0.00146091 | BGT |
| **McVSA1** | SRR5788230 | 99.998886 | 0.001113 | BGT |
| **McVSA1** | SRR5788233 | 99.99854 | 0.00145187 | BGT |
| **McVSA1** | SRR5788283 | 99.99851 | 0.00148699 | BGT |
| **McVSA1** | SRR5788282 | 99.99712 | 0.00287704 | BGT |
| **McVSA1** | SRR5788281 | 99.996635 | 0.00336963 | BGT |
| **McVSA1** | SRR5788284 | 99.99817 | 0.00182955 | BGT |
| **McVSA1** | SRR5788285 | 99.99818 | 0.00182364 | BGT |
| **McVSA1** | SRR5788286 | 99.99813 | 0.00186652 | BGT |
| **McVSA1** | SRR5788288 | 99.998695 | 0.00130245 | BGT |
| **McVSA1** | SRR5788318 | 99.9988 | 0.00119912 | BGT |
| **McVSA1** | SRR5788374 | 99.99895 | 0.00105563 | BGT |
| **McVSA1** | SRR5788430 | 99.99832 | 0.00168226 | BGT |
| **McVSA1** | SRR5788431 | 99.99872 | 0.00127943 | BGT |
| **McVSA1** | SRR5788436 | 99.99881 | 0.00118995 | BGT |
| **McVSA1** | SRR5788435 | 99.998566 | 0.00142894 | BGT |
| **McVSA1** | SRR5788429 | 99.998055 | 0.00194779 | BGT |
| **McVSA1** | SRR9178106 | 99.99787 | 0.00212924 | HOT |
| **McVSA1** | SRR9178213 | 99.998184 | 0.00181834 | HOT |
| **McVSA1** | SRR9178292 | 99.99803 | 0.00196652 | HOT |
| **McVSA1** | SRR9178368 | 99.99813 | 0.00186345 | HOT |
| **McVSA1** | SRR9178358 | 99.998146 | 0.00185231 | HOT |
| **McVSA1** | SRR9178335 | 99.997986 | 0.00201276 | HOT |
| **McVSA1** | SRR9178205 | 99.99348 | 0.00652092 | HOT |
| **McVSA1** | SRR9178320 | 99.994 | 0.00599536 | HOT |
| **McVSA1** | ERR594385 | 99.995705 | 0.00279515 | Tara |
| **McVSA1** | ERR594392 | 99.965965 | 0.01247225 | Tara |
| **McVSA1** | ERR594414 | 99.921646 | 0.03111607 | Tara |
| **McVSA1** | ERR594382 | 99.92059 | 0.03167623 | Tara |
| **McVSA1** | ERR594361 | 99.9567 | 0.01601857 | Tara |
| **McVSA1** | ERR594359 | 99.95748 | 0.01562878 | Tara |
| **McVSA1** | ERR594389 | 99.98083 | 0.00426101 | Tara |
| **McVSA1** | ERR594362 | 99.98643 | 0.00300507 | Tara |
| **McVSA1** | ERR599350 | 99.99125 | 0.00332009 | Tara |
| **McVSA1** | ERR599374 | 99.99579 | 0.00117695 | Tara |


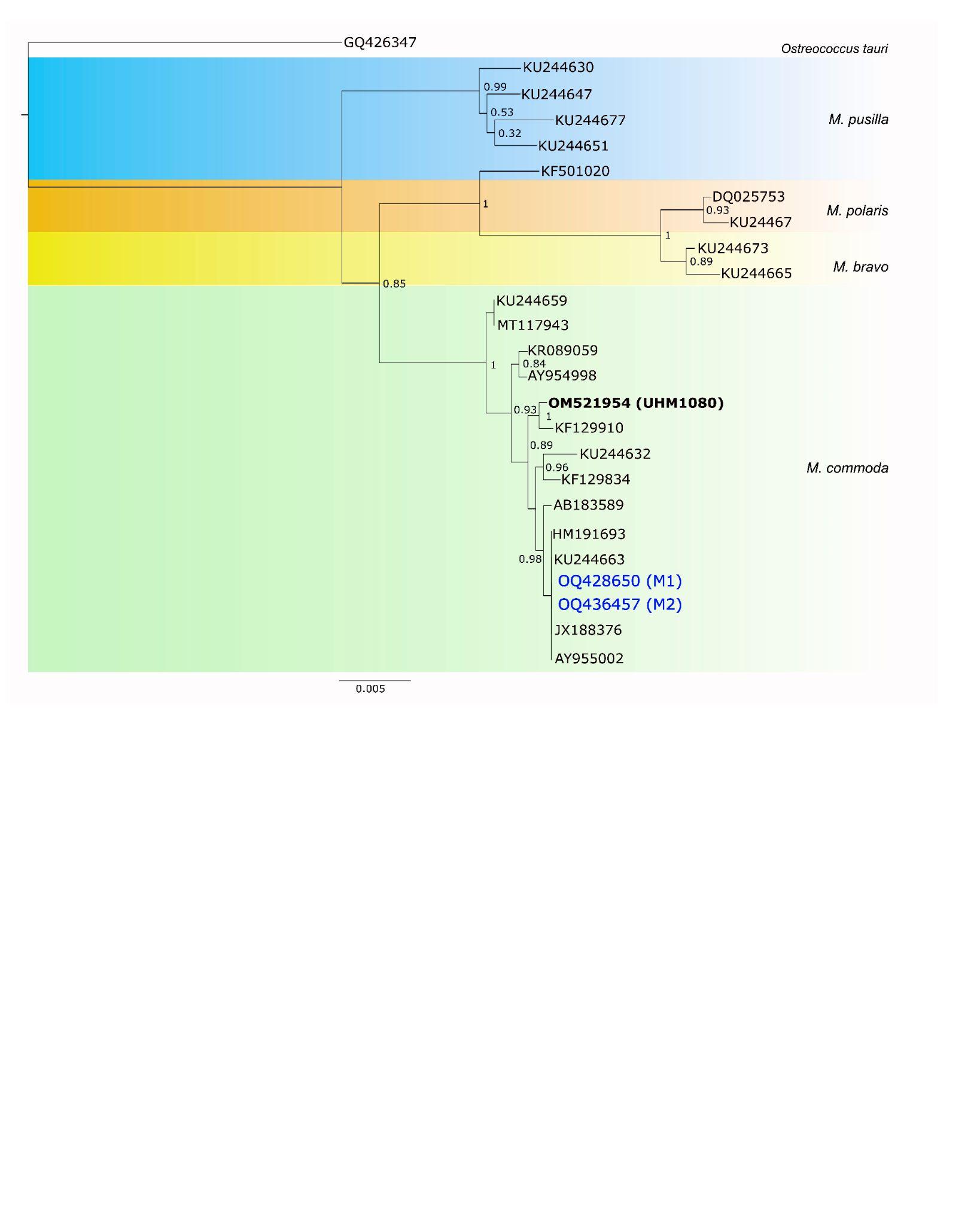


**Supplementary Figure S1**. A phylogenetic tree of Mamiellales. The tree is based on trimmed alignments of partial 18S rRNA genes from the two *Micromonas* strains used in this study (M1 and M2), a *Micromonas* strain from the pelagic Station ALOHA (UHM1080), and related sequences found using NCBI’s BLAST tool. Alignments were created and trimmed with Geneious 11.1 default alignment tool and processed through FastTree using approximately-maximum-likelihood. Node support values reflect FastTree local support values derived from the Shimodaira-Hasegawa test. Figure reproduced with permission from Bedi de Silva *et al*. (2024) Environ. Microbiol 26(8) e16686,


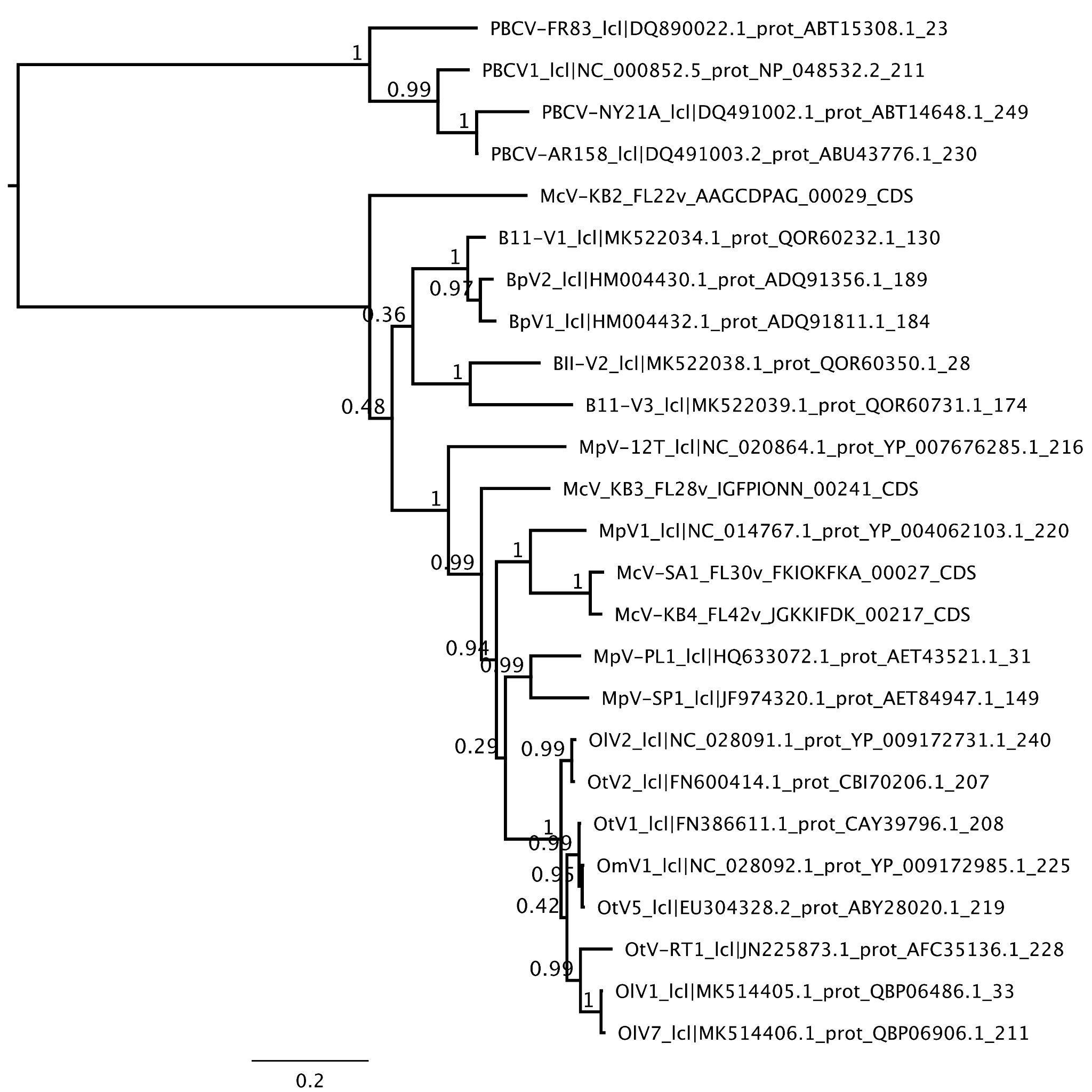


**Supplementary Figure S2.** Prasinovirus and chlorovirus species tree based on the polB orthogroup. Tree was created using FastTree, scale bar represents substitutions per site.


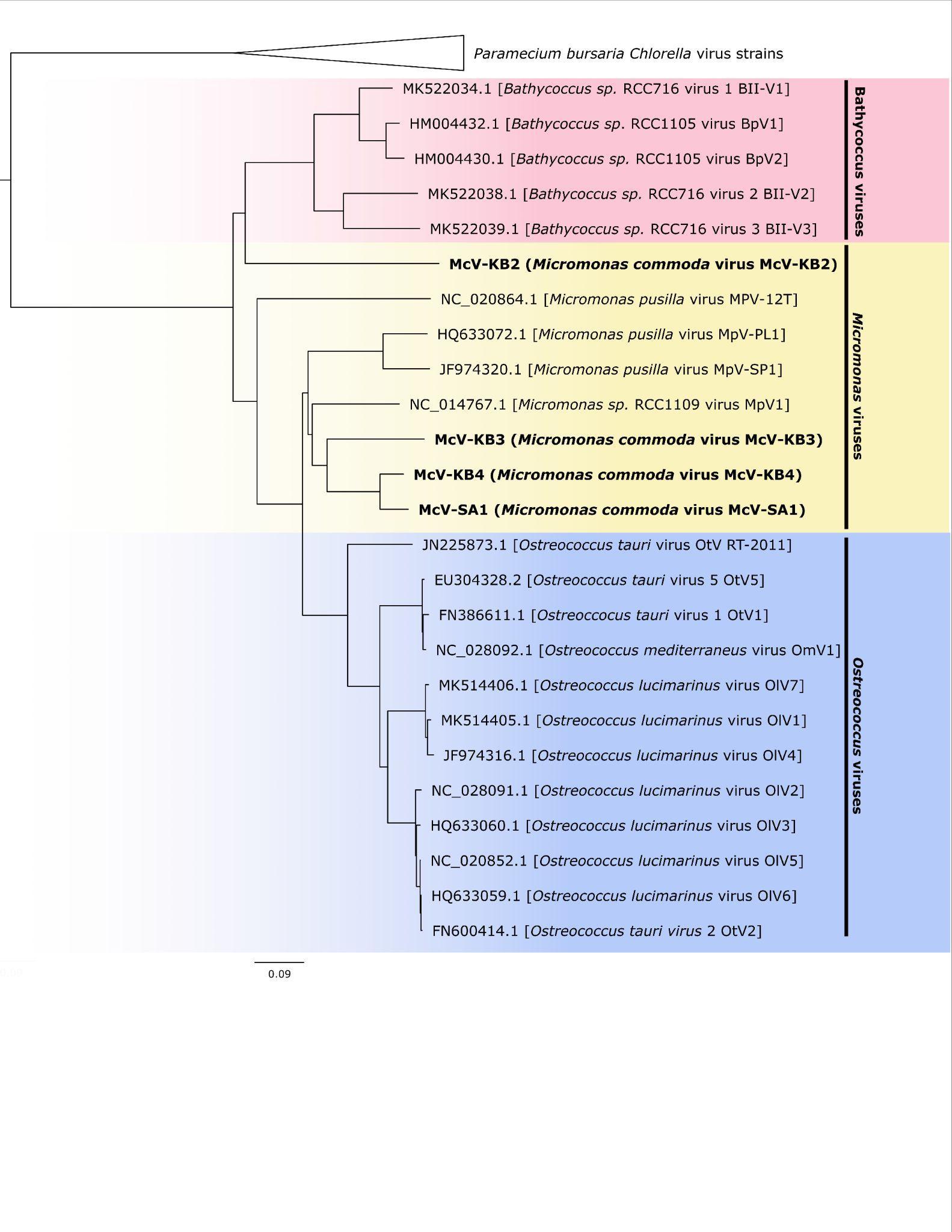


**Supplementary Figure S3**. STAG-generated species tree using the 26 orthogroups that are possessed by all prasinoviruses and chloroviruses genomes used in our OrthoFinder analysis. STAG bipartition support values are not available for datasets with fewer than 100 shared orthogroups. Scale bar indicates substitutions per site.

*
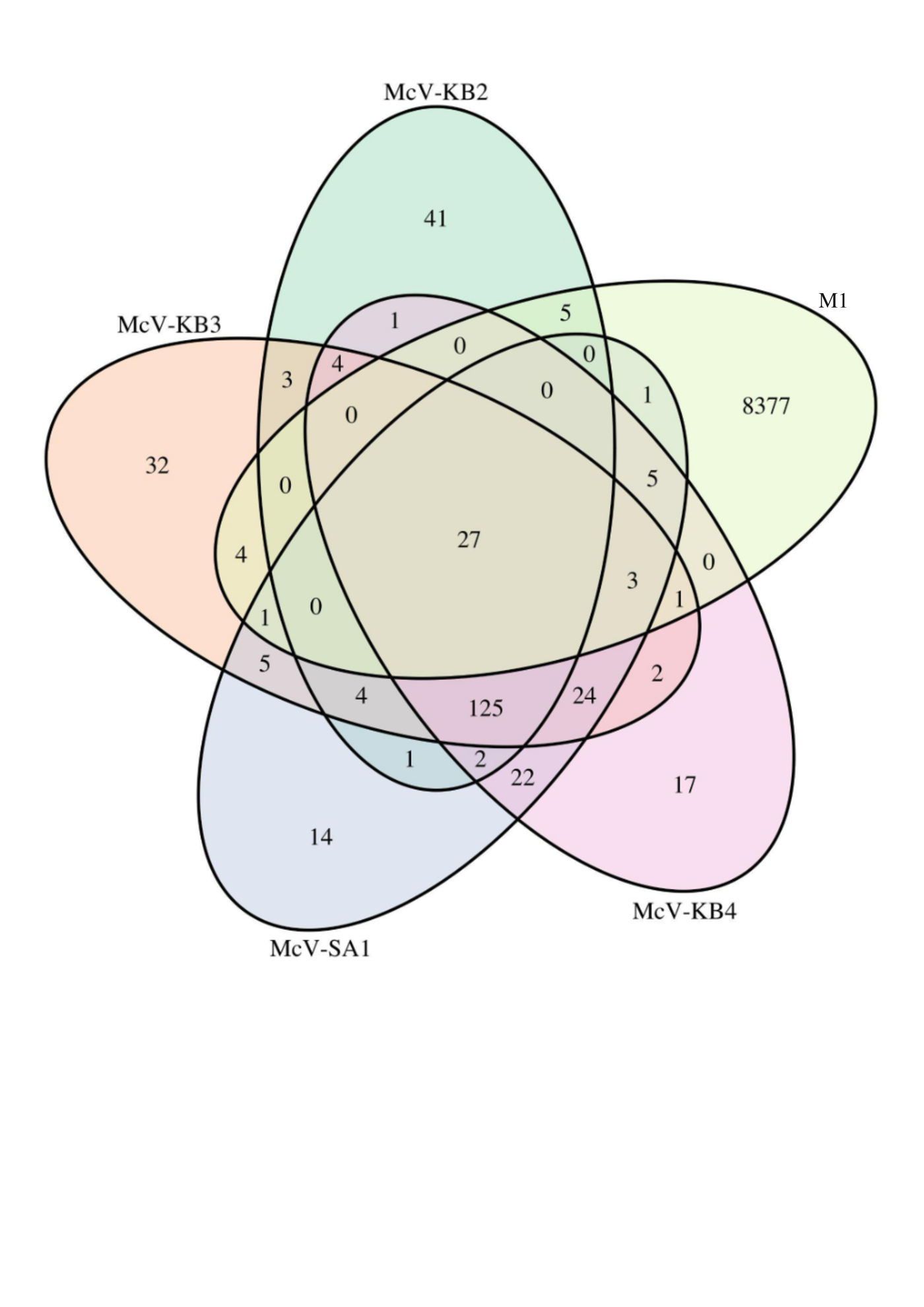
*

**Supplementary Figure S**4**.** Venn Diagram of the number of orthogroups shared between the four HiMcVs and host M1.

**
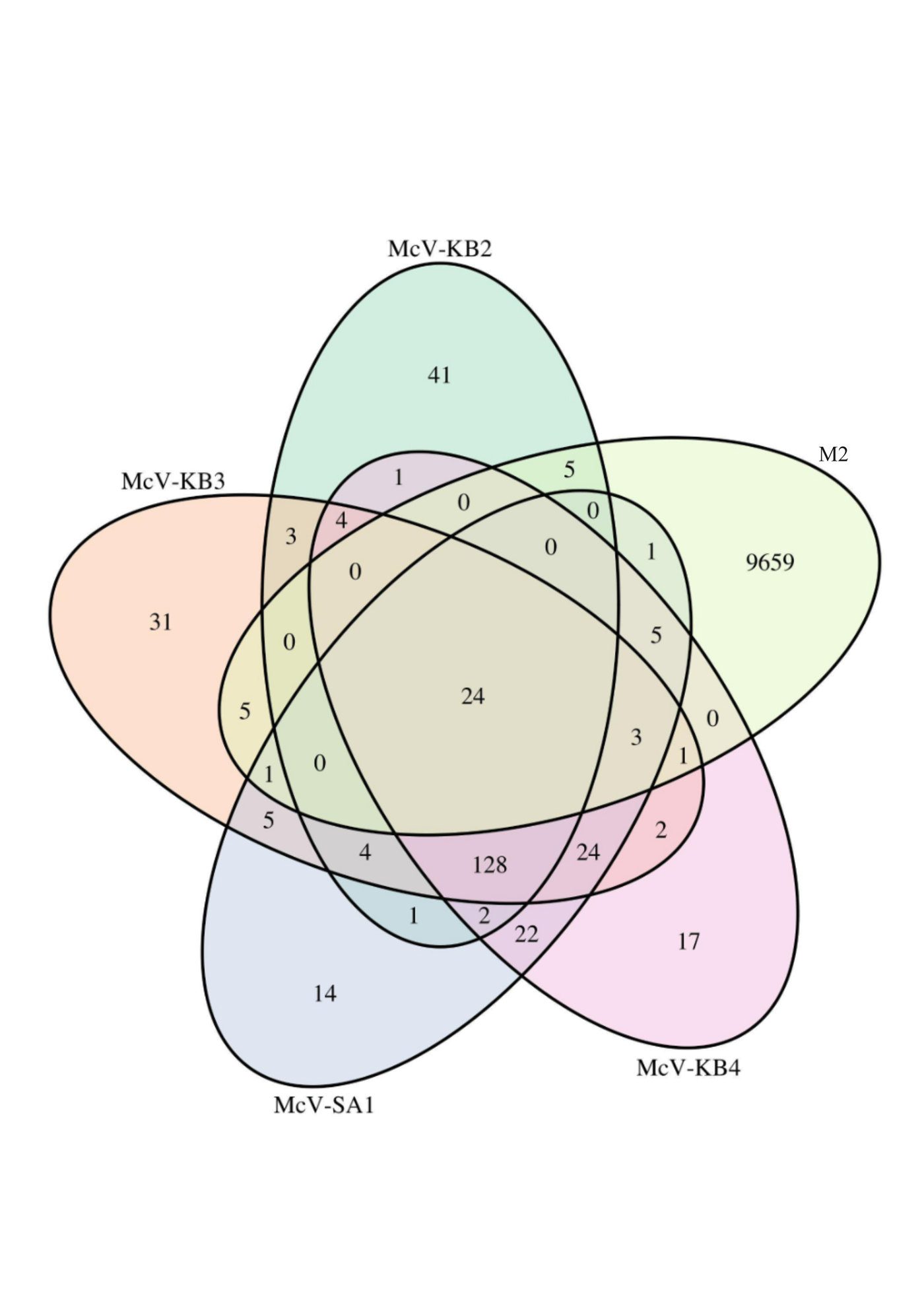
**

**Supplementary Figure S5.** Venn Diagram of the number of orthogroups shared between the four HiMcVs and host M2. Clustering analysis was conducted with OrthoFinder.


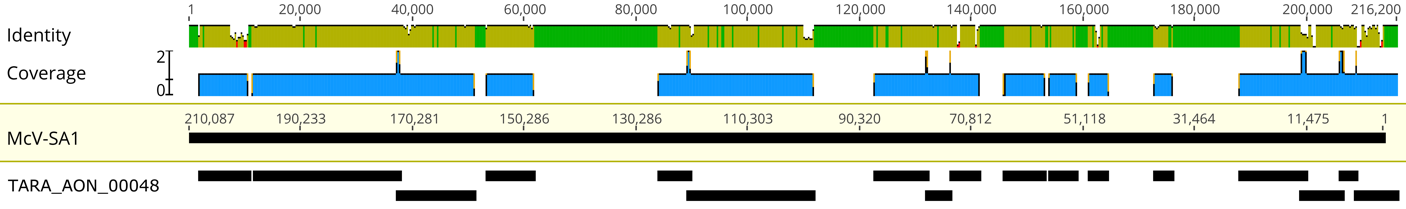


**Supplementary Figure S6.** Contigs from GVMAG TARA_AON_NCLDV_00048 mapped to McV-SA1. Coverage was 71%, with an average 92% nucleotide identity. Areas with no coverage are not included in calculations of average percent identity. Note that areas with no coverage are represented with gaps in the coverage graph but have values of 100% in the identity graph. Mapping of contigs was conducted in Geneious 11.1.
